# The Cross Talk Between Psoriasis, Obesity, and Dyslipidemia: A Meta-Analysis

**DOI:** 10.7759/cureus.49253

**Published:** 2023-11-22

**Authors:** Hyder Mirghani, Abdulaziz Talal Altemani, Sarah Talal Altemani, Juri Ahmad A Alhatlani, Naser Mansour I Alsulaimani, Deemah Salem A AlHuraish, Ali Hussein A Al Mudhi, Waad Jamal R Ghabban, Ahmed H Alanazi, Bander Ahmed Alamrani

**Affiliations:** 1 Internal Medicine, Faculty of Medicine, University of Tabuk, Tabuk, SAU; 2 Dermatology, King Fahad Specialist Hospital, Ministry of Health, Tabuk, SAU; 3 Dermatology, Faculty of Medicine, University of Tabuk, Tabuk, SAU; 4 Medicine, Faculty of Medicine, Qassim University, Qassim, SAU; 5 Medicine, Faculty of Medicine, King Abdulaziz University, Jeddah, SAU; 6 Medicine, College of Medicine, Imam Abdulrahman Bin Faisal University, Dammam, SAU; 7 General Practice, Dammam Medical Complex, Ministry of Health, Dammam, SAU; 8 Laboratory, King Khalid Hospital, Ministry of Health, Tabuk, SAU; 9 Internal Medicine, King Salman Armed Forces Hospital, Ministry of Defense, Tabuk, SAU; 10 Otorhinolaryngology, King Fahad Specialist Hospital, Ministry of Health, Tabuk, SAU

**Keywords:** comorbidity, meta-analysis, psoriasis, obesity, dyslipidemia

## Abstract

Although psoriasis is a multi-organ disease, it is usually managed as a skin disease, ignoring its associated serious comorbidities. This meta-analysis aimed to investigate the relationship between psoriasis, dyslipidemia, and obesity. Two authors independently searched three databases (PubMed, Medical Literature Analysis and Retrieval System Online (MEDLINE), The Cochrane Library, and Google Scholar). The search was set for articles published in the English language during the period from January 2013 to August 2023. The keywords "psoriasis", "hypercholesterolemia", "dyslipidemia", "low-density lipoproteins", "high body mass index", and "obesity", were used. Out of the 145 full texts reviewed, only seven studies fulfilled the inclusion and exclusion criteria (773,761 participants and 196,593 events). Psoriasis was associated with dyslipidemia and obesity (odds ratio (OR)=1.63, 95% CI: 1.42-1.88 and OR=1.70, 95% CI: 1.43-2.02), respectively, with significant heterogeneity (98% and 97%, respectively). Dyslipidemia and obesity were significant psoriasis comorbidities; a broader approach, viewing psoriasis as a multi-organ disease, is recommended for optimal treatment and outcomes.

## Introduction and background

Psoriasis is a multi-system inflammatory disorder. In addition to the skin, it affects various organs, including the cardiovascular, renal, and gastrointestinal systems. The disease is also associated with mood disorders, malignancy, and infections [1]. The disease is immune-mediated and linked to various components of the metabolic syndrome, including diabetes mellitus and insulin resistance, atherogenic dyslipidemia, high blood pressure, central adiposity, and metabolic-associated fatty liver disease [2]. The prevalence of metabolic syndrome among patients with psoriasis ranged from 20% to 50%, depending on severity [3]. Psoriasis and metabolic syndrome were shown to share the same metabolic pathways, genetic factors, and pathogenesis. The above observations imply that systemic chronic use of psoriasis treatment should be cautiously used to avoid the deterioration of coexisting metabolic diseases [1]. Obesity is a common multifactorial disease; the disease is on the rise globally, and currently, one-third of the population is affected [4]. Therefore, patients with psoriasis are candidates for obesity screening and weight reduction strategies to avoid serious consequences.

Previous literature showed the benefit of weight reduction on psoriasis severity [5]. Importantly, a higher body mass index is a factor in a low response to biological therapies [6]. Various studies have shown an association between psoriasis and dyslipidemia. Salihbegovic et al. [7] concluded the association between psoriasis, hypertriglyceridemia, and high-density lipoproteins. Supporting the findings of Nakhwa et al. [8], plausible explanations might be inadequate physical activity and bad dietary habits in addition to proinflammatory cytokines' effects on lipid metabolism [9]. Furthermore, dyslipidemia might be secondary to psoriasis treatment with cyclosporine and acitretin [10]. Dyslipidemia is a serious disease and is associated with cardiovascular disease. Assessing the association between dyslipidemia and psoriasis is a meaningful consideration. Therefore, this meta-analysis aimed to assess the relationship between psoriasis, dyslipidemia, and obesity.

## Review

Materials and methods

Eligibility Criteria According to Population, Intervention, Comparison, Outcomes, and Study (PICOS) Design

The current meta-analysis was conducted in accordance with the Preferred Reporting Items for Systematic Reviews and Meta-Analyses (PRISMA) guidelines.

Study Design and Duration

This meta-analysis was completed in July and August of 2023.

Inclusion Criteria

We included cross-sectional studies, case-control studies, retrospective studies, and prospective cohorts. The studies were those that assessed the association between psoriasis, dyslipidemia, and obesity.

Exclusion Criteria

Case reports, case series, and studies on animals were excluded.

Outcome Measures

The outcome measures were the association between psoriasis, dyslipidemia, and obesity.

Literature Search Strategy

Two authors independently searched PubMed, Medical Literature Analysis and Retrieval System Online (MEDLINE), the first 100 articles in Google Scholar, and the Cochrane Library for articles published in the English language between January 2013 to August 2023. The keywords "psoriasis", "hypercholesterolemia", "dyslipidemia", "low-density lipoproteins", "high body mass index", and "obesity" were used. In addition, the references of the included studies were searched for relevant articles. We identified 209 studies and 103 remained after the removal of duplication. From them, 28 full texts were screened, and only seven studies were included in the final meta-analysis.

Data Extraction

**Figure 1:**
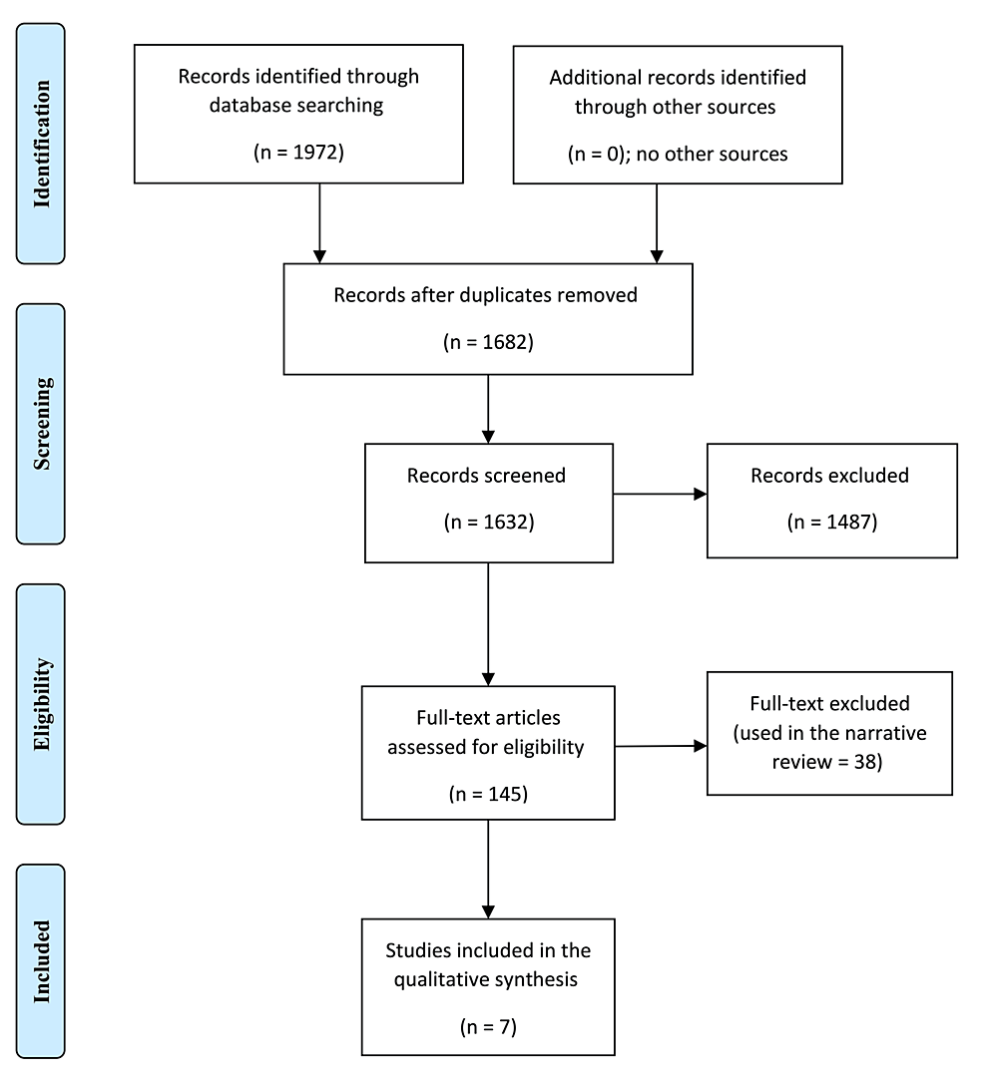
Studies on dyslipidemia and obesity among patients with psoriasis and control subjects selected as per PRISMA guidelines PRISMA: Preferred Reporting Items for Systematic Reviews and Meta-Analyses

A datasheet was used to extract the author's name, year of publication, country of publication, details on dyslipidemia and obesity among cases and controls, age, and sex of the participants (Tables 1-2 and Figure 1).

**Table 1:** Dyslipidemia among patients with psoriasis and control subjects (the data have been represented as percentages)

Author	Methods	Psoriasis	Controls	Results
Feldman et al. [11]	Retrospective	425/6868	351/1230	Significant, USA
Feldman et al. [12]	Retrospective	44489/114824	37526/114824	Significant, USA
Fernández-Armenteros et al. [13]	Cross-sectional	1978/6868	69374/398701	Significant, Spain
Kaine et al. [14]	Retrospective	5,038/14898	10412/35,037	Significant, USA
Kampe et al. [15]	Cross-sectional	2778/7249	22459/72,490	Significant, Slovakia
Lee et al. [16]	Retrospective	816/7245	606/7245	Significant, USA
Sun et al. [17]	Retrospective	207/307	134/613	Significant, China

**Table 2:** Obesity among patients with psoriasis and control subjects (the data have been represented as percentages)

Author	Methods	Psoriasis	Controls	Results
Feldman et al. [11]	Retrospective	53/1230	40/1230	Significant, USA
Feldman et al. [12]	Retrospective	7598/114824	5069/114824	Significant, USA
Fernández-Armenteros et al. [13]	Cross-sectional	2314/6868	112035/398701	Significant, Spain
Kaine et al. [14]	Retrospective	2,365/14,898	4,452/35,037	Significant, USA
Kampe et al. [15]	Cross-sectional	777/7249	5023/72,490	Significant, Slovakia
Lee et al. [16]	Retrospective	209/7245	187/7245	Significant, USA
Sun et al. [17]	Retrospective	164/307	47/613	Significant, China

Risk of Bias Assessment

The Newcastle-Ottawa Scale assessed the quality of the included studies.

Statistical Analysis

The RevMan system (The Cochrane Collaboration, London, UK) was used for data analysis, and the DerSimonian and Laird approach was applied. Seven studies were pooled, and all were observational. The dichotomous data were entered manually, and random effects were applied depending on the significance. A P-value of <0.05 was considered significant.

Results

**Figure 2:**
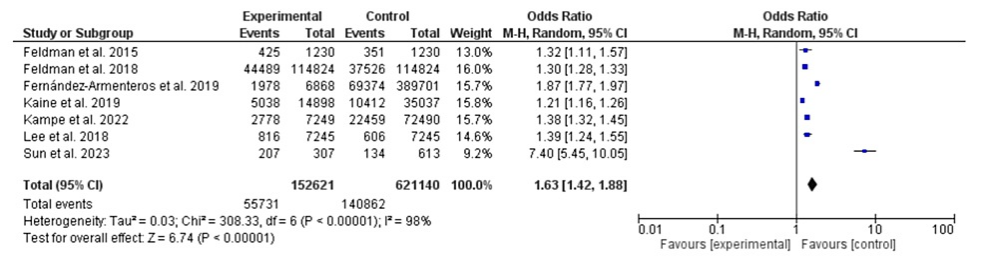
Dyslipidemia among patients with psoriasis and control subjects Feldman et al., 2015: [11]; Feldman et al., 2018: [12]; Fernández-Armenteros et al., 2019: [13]; Kaine et al., 2019: [14]; Kampe et al., 2022: [15]; Lee et al., 2018: [16]; Sun et al., 2023: [17]

This meta-analysis included seven studies, four from the United States of America, two from Europe, and one from Asia. The majority of the included studies were retrospective (five), in addition to two cross-sectional studies. The studies included 773,761 participants and 196,593 events. Psoriasis was associated with dyslipidemia (odds ratio (OR)=1.63, 95% CI: 1.42-1.88, the Chi-Square was 308.33, and the P-value was <0.001. Substantial heterogeneity was found to be 98%, and the P-value for heterogeneity was <0.001 (Figure 2).

**Figure 3:**
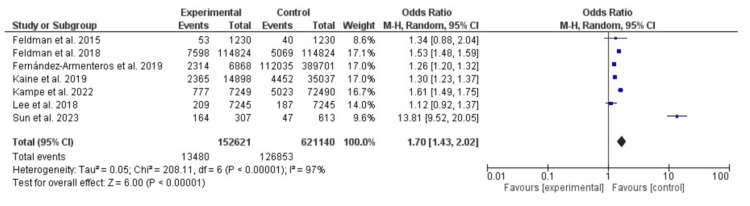
Obesity among patients with psoriasis and control subjects Feldman et al., 2015: [11]; Feldman et al., 2018: [12]; Fernández-Armenteros et al., 2019: [13]; Kaine et al., 2019: [14]; Kampe et al., 2022: [15]; Lee et al., 2018: [16]; Sun et al., 2023: [17]

Psoriasis was also associated with obesity (OR=1.70, 95% CI: 1.43-2.02, the Chi-square was 308.33, and the P-value was <0.001. Substantial heterogeneity was found at 97%, with a P-value for heterogeneity <0.001 (Figure 3).

Discussion

In the current meta-analysis, psoriasis was associated with dyslipidemia and obesity, respectively (OR=1.63, 95% CI: 1.42-1.88, and OR=1.70, 95% CI: 1.43-2.02). The present findings are in line with Choudhary et al. [18], who published a meta-analysis and found similar findings. The current findings support Miller et al. [19], who found a strong association between psoriasis, obesity, dyslipidemia, and other components of the metabolic syndrome. Interestingly, high body mass index and female sex were strong predictors of biological discontinuation. The authors explained their findings through side effects and ineffectiveness [20]. Therefore, screening for obesity is an effective tool for biological treatment adherence and optimization of treatment. The casual relationship between psoriasis and obesity might be explained by the disturbed function of the skin barrier and lymphatic system [21,22].

Vitamin D deficiency has been linked to an increased risk of developing psoriasis, obesity, and dyslipidemia. This is due to the role that vitamin D plays in regulating the immune system and inflammation, both of which are key factors in the development and progression of these conditions. Therefore, it is important to take into account the potential impact of vitamin D deficiency when studying the relationship between psoriasis, obesity, and dyslipidemia [23, 24].

Stress has also been identified as a potential confounder in the relationship between these three conditions. Chronic stress has been shown to exacerbate psoriasis and contributes to the development of obesity and dyslipidemia. This is thought to be due to the impact of stress on hormone levels, inflammation, and immune function. Therefore, it is important to consider the potential influence of stress when studying the cross talk between psoriasis, obesity, and dyslipidemia [25].

In addition, tumor necrosis factor and interleukin-6 secretion mediated the inflammatory response [26, 27]. High leptin levels are important factors in keratinocyte proliferation, and adiponectin showed an anti-inflammatory response [28, 29]. The above findings imply that targeting the cytokine level might be an effective preventive and treatment strategy for patients with psoriasis. In addition, the adoption of healthy lifestyles among obese patients with psoriasis cannot be overlooked; weight loss was found to decrease psoriasis skin lesions and joint disease [30]. Importantly, glucagon-like peptide-1 (GLP-1) agonists were found to be effective in reducing psoriasis area, severity index, and fasting plasma glucose among patients with psoriasis and diabetes mellitus [31]. Therefore, GLP-1 agonists are a good option for patients with psoriasis, high body mass index, and diabetes. The association of psoriasis with dyslipidemia raises concerns about certain medications; cyclosporine and acitretin should be used with caution to avoid their lipid profiles [32, 33]. Close monitoring of serum lipid and body mass index is important among patients with psoriasis to avoid cardiovascular disease and improve response and adherence to therapy. Treatment of obesity and dyslipidemia was shown to prevent disease exacerbation in recent animal studies [34]. An interesting recent study found that high-density lipoprotein/triglyceride levels predict psoriasis genetically, in particular among young females [35]. The small number of studies included and their observational nature limited the current study, and significant heterogeneity was observed.

Overall, the consideration of confounders such as vitamin D deficiency and stress is essential for advancing our understanding of the cross talk between psoriasis, obesity, and dyslipidemia. By carefully accounting for these factors in research and clinical practice, we can work towards improving the management and outcomes of individuals affected by these interconnected conditions [23, 25].

## Conclusions

Patients with psoriasis were more obese compared to those without the disease; screening for obesity is vital to avoid its deleterious consequences. Lifestyle modifications are suggested for patients with psoriasis for a better outcome. In addition, dyslipidemia is more common among patients with psoriasis; screening for obesity, overweight, and other parameters of the metabolic syndrome is highly recommended. Early treatment of comorbid disorders is vital to avoiding cardiovascular disease. Certain medications with negative effects on serum lipids should be used cautiously.
